# Optimizing the Diagnosis of Microcytic Hypochromic Anemia: A Comparative Evaluation of Erythrocyte and Reticulocyte Parameters

**DOI:** 10.7759/cureus.69244

**Published:** 2024-09-12

**Authors:** Savitri M Nerune, Sohan Rao H. R., K. Pallavi, Amogh P Lavate, Sayandeep K Das, Sajal Pagi

**Affiliations:** 1 Pathology, Shri B.M. Patil Medical College Hospital and Research Centre, Bijapur Lingayat District Educational (BLDE) Association (Deemed to be University), Vijayapura, IND; 2 Pathology, Central Referral Hospital, Sikkim Manipal Institute of Medical Sciences, Gangtok, IND; 3 Pathology, GSL Medical College, Rajahmundry, IND

**Keywords:** bessman index, england and fraser index, hematological indices, iron deficiency anemia (ida), microcytic hypochromic anemia, red cell distribution width (rdw), β-thalassemia

## Abstract

Background

Anemia is a widespread, worldwide hematological disorder, with iron deficiency anemia (IDA) and thalassemia trait (TT) being two frequently encountered forms. For effective management, it is vital to distinguish between these two conditions. Existing diagnostic methods have some limitations due to their inaccessibility and high cost. This study aims to evaluate novel hematological indices for distinguishing IDA from TT, offering potential improvements in diagnostic accuracy and patient care.

Objective

To compare the efficacy of novel hematological indices in differentiating IDA from TT, and to assess their sensitivity and specificity in clinical practice.

Methods

This cross-sectional observational study included 80 anemia patients (40 diagnosed cases of IDA and 40 of TT each). Hematological parameters were measured using the Sysmex XN-1000 analyzer (Sysmex Corporation, Kobe, Japan), and statistical analysis was conducted using the Mann-Whitney test, Pearson correlation coefficient (r), and correlation matrix.

Results

Significant differences were observed between IDA and TT patients in the indices studied. The Green and King Index demonstrated high diagnostic accuracy, with a sensitivity of 90% and specificity of 82%. The red cell distribution width (RDW) Index exhibited similar diagnostic performance, with a sensitivity of 90% and specificity of 77%. The England and Fraser Index showed a sensitivity of 85% and specificity of 80%, with a large effect size (r = -0.60). The correlation matrix revealed strong associations between key parameters, including a negative correlation between hemoglobin and RDW Index (r = -0.61) and a positive correlation between mean corpuscular volume (MCV) and Shine and Lal Index (r = 0.82). On the other hand, our study found that indices like Shine and Lal and Srivastava had limited diagnostic value, with smaller effect sizes (r = 0.28 and r = -0.005, respectively).

Conclusion

Hematological indices, such as the Green and King, RDW, and England and Fraser indices, show strong potential for differentiating IDA from TT, with high sensitivity, specificity, and large effect sizes. The correlation matrix further supports the diagnostic utility of these indices. These tools can enhance diagnostic precision in resource-limited settings and improve patient outcomes.

## Introduction

Iron deficiency anemia (IDA) and thalassemia trait (TT) are the most common forms of anemia, affecting millions of children worldwide [1]. According to recent estimates by the World Health Organization (WHO), anemia affects about 40% of children, 37% of pregnant women, and 30% of women across the globe [2,3]. The etiology of microcytic hypochromic anemia includes nutritional deficiencies, inflammation, and genetic disorders [4]. IDA predominantly affects women of childbearing age and children [5]. This is a well-known problem in society, particularly in rural areas, where it mostly affects young children, posing a major public health issue [6].

Differentiating microcytic hypochromic anemia is essential due to the differing prognoses and treatment approaches. Initially, with the help of automated blood analyzers, red blood cell (RBC) parameters are calculated, and the microscopic blood film is analyzed. Therefore, there is a need for more laboratory tests, since the two conditions are almost similar. The diagnosis of IDA is obtained and confirmed by measuring serum iron, total iron-binding capacity (TIBC), and serum ferritin levels, while the diagnosis of β-TT is made by measuring elevated HbA2 levels with the help of high-performance liquid chromatography (HPLC). Although these tests are very accurate, they are also very costly, time-consuming, and hardly available in poorer, resource-limited regions [7,8].

Recent data from the WHO highlight the necessity for accessible and affordable diagnostic tools for microcytic hypochromic anemia. One such diagnostic tool is the evaluation of erythrocyte and reticulocyte parameters, which can provide insight into the body’s erythropoietic activity and help classify the diverse types of anemia. Automated hematology analyzers, like the Sysmex XN-1000 (Sysmex Corporation, Kobe, Japan), can be used to determine these parameters, which can be effective for identifying functional iron deficiency and ruling out TT from IDA [9].

The aim of the present study was to compare the efficacy of IDA and TT by evaluating novel erythrocyte and reticulocyte parameters. The indices employed in our study included the England and Fraser Index, which, based on RBC parameters, distinguishes between IDA and TT - negative values indicating TT and positive values indicating IDA [10]. The Shine and Lal Index differentiates IDA and TT by evaluating mean corpuscular volume (MCV) and RBC count, with values less than 1530 indicating β-TT [11]. The Srivastava Index combines multiple parameters for differentiation, with values less than 3.8 indicating IDA [12]. The Mentzer Index differentiates microcytic anemia based on the ratio of MCV to RBC count, with values less than 13 indicating IDA [13]. The Green and King Index distinguishes between IDA and TT by employing a formula in which the MCV is squared and then multiplied by the red cell distribution width (RDW). The product is divided by the amount of hemoglobin (Hb) present and then multiplied by 1000, with values less than 72 being indicative of β-TT [14]. The RDW index was also used for differentiation, with values less than 220 indicating β-TT [14]. The Bessman Index, which distinguishes the two conditions, has a value greater than 15 suggesting IDA [15]. Examining all these parameters in combination may help provide a way of effectively screening, diagnosing, and managing microcytic hypochromic anemia.

## Materials and methods

Study setting and period

This cross-sectional observational study was carried out in a tertiary care hospital. The study included a cohort of 80 patients, divided into 40 each for IDA and TT, from February 1, 2023, to January 31, 2024.

Inclusion and exclusion criteria

The inclusion criteria for this study included all newly diagnosed cases of IDA and TT. There were no exclusion criteria for this study.

Study design

Blood samples were collected from each participant for detailed hematological and biochemical investigations. Specifically, 2 mL of blood was drawn into ethylene diamine tetraacetic acid (EDTA) vacutainers for the assessment of RBC and reticulocyte parameters using the Sysmex XN-1000 hematology analyzer. HPLC was also carried out to determine HbA2 levels, confirming the diagnosis of TT. Additionally, 2 mL of blood was collected in plain vacutainers for the measurement of serum iron, serum ferritin, and TIBC to confirm the diagnosis of IDA.

The erythrocyte and reticulocyte parameters measured in this study were RBC count, Hb, MCV, mean corpuscular hemoglobin (MCH), mean corpuscular hemoglobin concentration (MCHC), RDW, reticulocyte count, and reticulocyte production index (RPI).

Statistical analysis

The statistical analyses were performed using IBM SPSS Statistics for Windows, Version 28 (Released 2021; IBM Corp., Armonk, NY, USA) and Python (Version 3.11). The normality of the data distributions was assessed using the Shapiro-Wilk test. The results indicated a deviation from normality, warranting the use of the non-parametric Mann-Whitney U test for comparing hematological indices between the IDA and TT groups. A p-value of <0.05 was considered statistically significant. Sensitivity and specificity analyses were performed to evaluate the diagnostic performance of the indices, with the Youden Index used to calculate the optimal cut-off values.

Effect sizes were calculated using Pearson’s r, derived from the Mann-Whitney U test, to quantify the magnitude of differences between the groups. Additional correlation analyses were performed to explore the relationships between various hematological indices.

## Results

**Figure 1:**
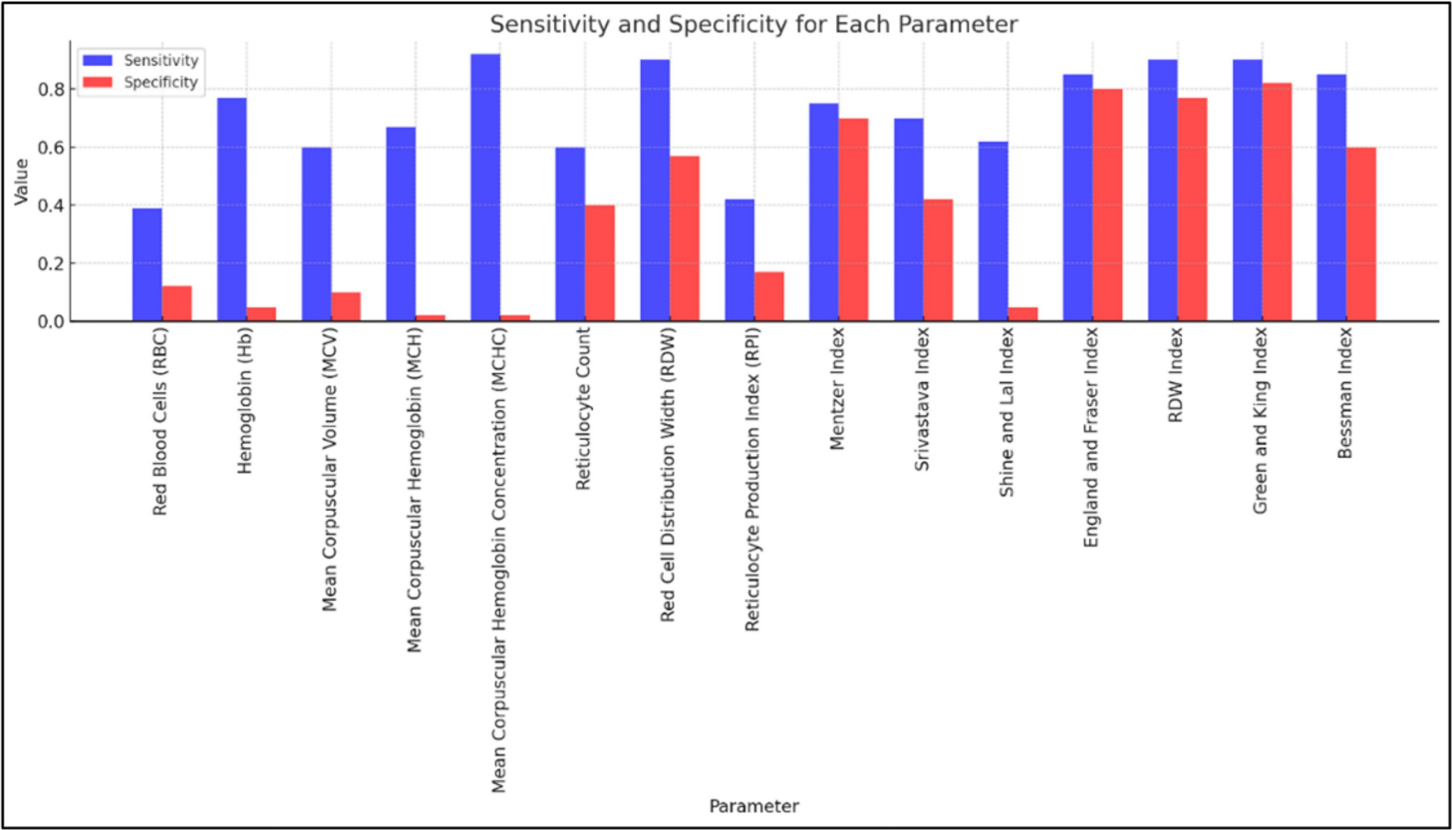
Sensitivity and specificity of the parameters

The findings from our study revealed a significant distinction between IDA and TT using various hematological indices. Each index was analyzed based on its p-value, sensitivity, and specificity to determine its diagnostic utility (Figure 1). The indices assessed and the statistical findings are laid out in Table 1.

**Table 1:** The parameters assessed and their statistical values across IDA and TT groups *statistically significant IDA: Iron deficiency anemia; TT: Thalassemia trait

Parameter	Thalassemia (mean ± SD)	IDA (mean ± SD)	Mann-Whitney test	p-value	Sensitivity	Specificity
Red blood cells (RBCs)	5.23 ± 1.14	3.96 ± 0.90	277	<0.001*	39%	12%
Hemoglobin (Hb)	10.26 ± 2.14	6.50 ± 1.54	117.5	<0.001*	77%	5%
Mean corpuscular volume (MCV)	63.61 ± 5.24	60.71 ± 7.59	557	0.019*	60%	10%
Mean corpuscular hemoglobin (MCH)	20.98 ± 6.52	16.66 ± 3.33	301	<0.001*	67%	2%
Mean corpuscular hemoglobin concentration (MCHC)	31.22 ± 2.42	27.19 ± 2.58	182	<0.001*	92%	2%
Reticulocyte count	1.58 ± 1.12	1.57 ± 0.93	769	0.765	60%	40%
Red cell distribution width (RDW)	18.00 ± 2.74	21.77 ± 3.21	255	<0.001*	90%	57%
Reticulocyte production index (RPI)	0.70 ± 0.39	0.40 ± 0.29	317.5	<0.001*	42%	17%
Green and King index	79.98 ± 61.55	133.08 ± 55.99	163	<0.001*	90%	82%
RDW index	255.59 ± 244.77	358.31 ± 139.96	229	<0.001*	90%	77%
England and Fraser index	3.67 ± 12.85	20.86 ± 10.30	208	<0.001*	85%	80%
Bessman index	17.99 ± 2.74	21.77 ± 3.211	255	<0.001*	85%	60%
Mentzer index	13.51 ± 7.90	16.56 ± 6.11	428	<0.001*	75%	70%
Srivastava index	4.64 ± 3.78	4.55 ± 1.89	661	0.181	70%	42%
Shine and Lal index	869.55 ± 355.00	650.92 ± 297.69	420	<0.001*	62%	5%

From the present study, we found that the Green and King Index is a highly effective diagnostic tool, with a p-value of <0.001, a sensitivity of 90%, and a specificity of 82%. It is reliable for both identifying TT and excluding IDA, with the potential to be one of the best tools for clinical use. The RDW Index showed a statistically significant difference (p-value <0.001) and demonstrates high effectiveness, with a sensitivity of 90% and a specificity of 77%. The England and Fraser Index was also highly effective, with a p-value of <0.001, a sensitivity of 85%, and a specificity of 80%. These values indicate that it is a robust tool for distinguishing between TT and IDA, with high accuracy and reliability. The Bessman Index showed statistical significance (p-value <0.001), with a sensitivity of 85% and a specificity of 60%. While it is effective in detecting TT, the comparatively lower specificity suggests a higher rate of false positives; thus, it should be used alongside other indices to improve overall diagnostic accuracy. The Mentzer Index showed a statistically significant difference between TT and IDA, with a p-value of <0.001. It has a sensitivity of 75%, meaning it correctly identifies most TT cases, but with a specificity of 70%, there is a chance of false positives; hence, this should also be used in conjunction with other indices to ensure accuracy.

With a p-value of 0.181, the Srivastava Index does not show statistical significance. Along with a sensitivity of 70% and a low specificity of 42%, the Srivastava Index has a high rate of false positives. The Shine and Lal Index is statistically significant (p-value <0.001) but has a relatively low sensitivity of 62% and an extremely low specificity of 5%. This suggests that, while it can identify some cases of TT, it is not reliable for ruling out IDA due to the high rate of false positives.

The Green and King Index, RDW Index, and England and Fraser Index stand out for their reliability in diagnostic performance. In contrast, indices like the Srivastava Index and the Shine and Lal Index show limitations and should be used cautiously. These combined findings suggest that, while individual indices can provide valuable insights, the best diagnostic accuracy is achieved when multiple indices are used together, allowing for a more comprehensive assessment.

The effect sizes for various hematological indices were calculated to evaluate the practical significance of the differences between TT and IDA groups (Table 2).

**Table 2:** Effect size analysis of the parameters MCV: Mean corpuscular volume; RDW: Red cell distribution width; Hb: Hemoglobin

Parameters	r	Effect size
Hb	0.632932788	Large
MCV	0.070829974	None
Mentzer	-0.23218881	Small
Shine and Lal	0.282323866	Small
Srivastava	-0.00512752	None
England and Fraser	-0.6027659	Large
RDW	-0.51193579	Large
Green and King	-0.42635372	Medium
Bessman	-0.51193421	Large

The England and Fraser Index (r = -0.60) demonstrates the largest effect size, highlighting its strong ability to differentiate between IDA and TT. The RDW Index (r = -0.51) also shows a substantial effect, confirming its effectiveness in distinguishing between the two conditions. The Bessman Index shares a similar large effect size (r = -0.51), indicating its reliability in assessing red cell distribution variations.

The Green and King Index exhibits a medium effect size of r = -0.43, making it a valuable diagnostic tool, though slightly less powerful compared to the aforementioned indices. In contrast, the Mentzer Index and the Shine and Lal Index show smaller effect sizes (r = -0.23 and r = 0.28, respectively), suggesting they may be more effective when used alongside other indices rather than independently.

**Figure 2:**
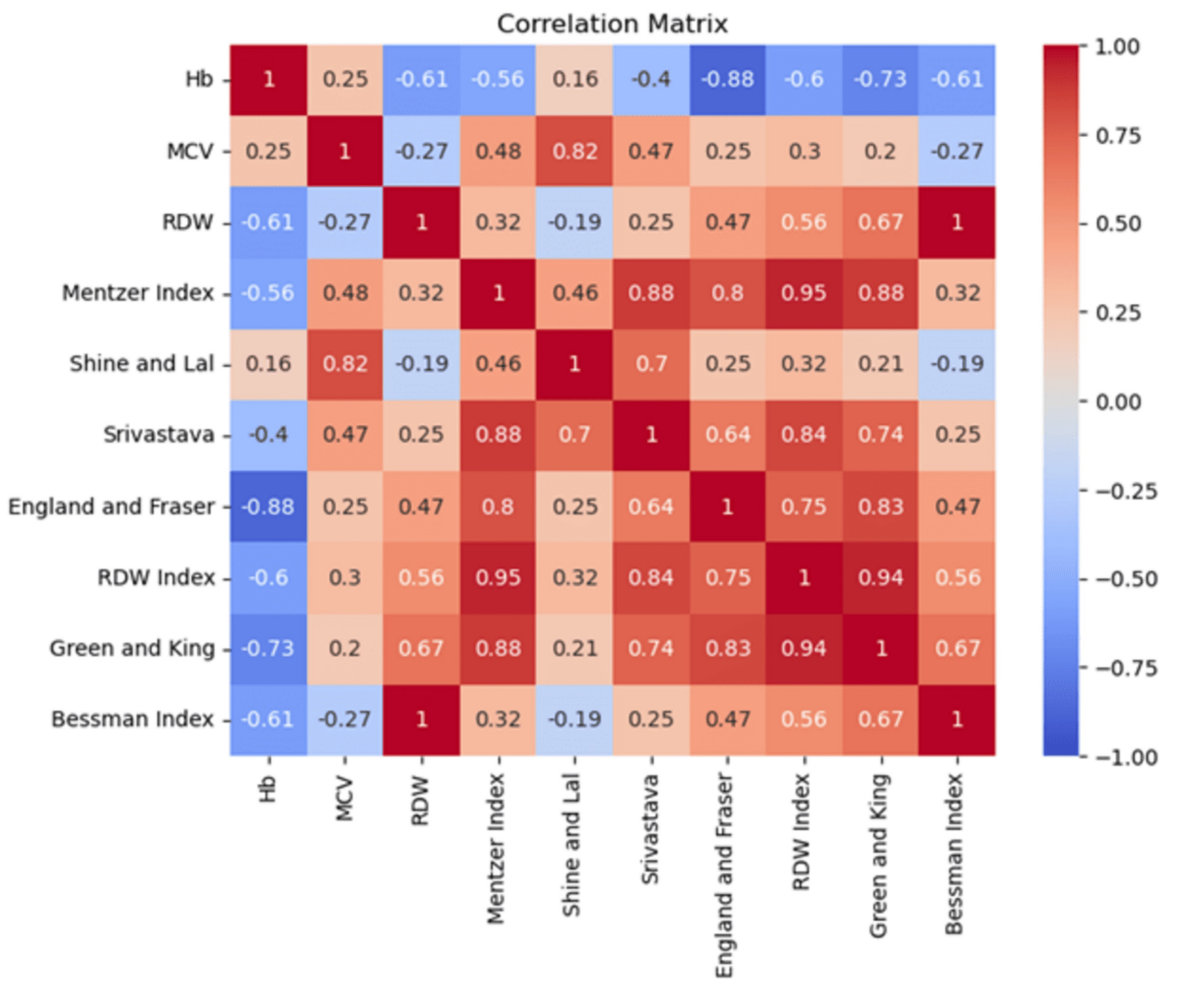
Correlation matrix of the key parameters form our study MCV: Mean corpuscular volume; RDW: Red cell distribution width; Hb: hemoglobin

The correlation matrix (Figure 2) shows some important associations between hematological indices in differentiating between IDA and TT. Hb has a negative correlation with RDW (-0.61), the Mentzer Index (-0.56), and the RDW Index (-0.60), which means that as Hb decreases, these indices increase, in concordance with the changes expected in these conditions. MCV has a high correlation with the Shine and Lal Index (r = 0.82), thus reinforcing its relation with the size of RBCs, a parameter important for diagnosing TT. RDW has a positive correlation with the RDW Index (r = 0.56) and the Green and King Index (r = 0.67), which confirms the role of RDW in identifying the variation in red cell size, helpful in differentiating between IDA and TT. The Mentzer Index correlates with the Srivastava Index (r = 0.88), the England and Fraser Index (r = 0.80), and the RDW Index (r = 0.95); this supports the ability of the Mentzer Index to distinguish these conditions. The England and Fraser Index correlates well with Hb (r = -0.88) and with the Mentzer Index (r = 0.80). The Bessman Index correlates well with RDW (r = 1.00) and has a moderate negative correlation with Hb (r = -0.61). Overall, the RDW Index (r = -0.51) and the Green and King Index (r = -0.67) emerge as highly dependable for distinguishing IDA from TT, with the England and Fraser Index and other indices providing valuable support. This underscores the need to employ multiple indices to arrive at the correct diagnosis.

## Discussion

It is essential to distinguish microcytic anemia due to the different prognoses and treatment strategies involved. The initial diagnosis entails the study of a microscopic blood film and the measurement of RBC indices. Additional examinations are required to distinguish between IDA and TT. To diagnose IDA, healthcare professionals rely on tests of blood iron levels, TIBC, and serum ferritin. On the other hand, TT is diagnosed by performing hemoglobin electrophoresis and noting high levels of HbA2. Several discriminant indices have been suggested to streamline the process of distinguishing between alternative possibilities, such as the Green and King Index, England and Fraser Index, and erythrocyte count [16]. However, only a few of these indices have sufficient sensitivity and specificity to establish a diagnosis [17-19].

The present study demonstrated that the Green and King Index, RDW Index, and England and Fraser Index were particularly effective in differentiating IDA from TT, with high sensitivity and specificity. These indices showed strong diagnostic value and could serve as reliable tools in resource-limited settings. The Bessman Index, while also effective, showed moderate specificity, suggesting it is best used in combination with other indices. The Mentzer Index and Shine and Lal Index, though useful, were less powerful and are recommended to be used in conjunction with other indices for a more accurate diagnosis.

The Green and King Index has been recognized in previous studies for its high sensitivity and specificity in differentiating IDA from TT. Zaghloul et al. found that the Green and King Index had a sensitivity of 85% and a specificity of 80% in a cohort similar to ours [20], which suggests that the Green and King Index is a reliable tool that can be confidently used in diverse populations and poor settings. Reis et al. reported that the RDW Index was particularly effective in distinguishing IDA from TT in cases where MCV was borderline, which aligns with the present findings [21]. We observed a sensitivity of 90% and a specificity of 77%, which validates its diagnostic utility as a valuable discriminant.

The England and Fraser Index, although less frequently discussed in the literature, has shown comparable diagnostic accuracy to other indices. A study by Wickramaratne and Wijewickrama found this index to be highly effective in differentiating TT from IDA, with sensitivity and specificity very similar to those found in our study (85% and 80%, respectively) [22]. Hence, the England and Fraser Index’s integration of multiple hematological parameters can make it a reliable tool, especially in settings where more expensive tests, like Hb electrophoresis, might not be available.

The Bessman Index, though not as widely studied, has been reported by Hoffmann et al. to be a useful supplementary tool in distinguishing between microcytic anemias [23]. Our study supports this, with the Bessman Index showing 85% sensitivity and 60% specificity, suggesting that it is most effective when used in combination with other indices.

Both the Mentzer Index and the Shine and Lal Index have been evaluated in numerous studies, often with mixed results. Tabassum et al. found the Mentzer Index to be moderately effective, similar to our findings, where it showed a sensitivity of 75% and a specificity of 70% [13]. The Shine and Lal Index, on the other hand, has been shown to have low specificity in previous studies, which aligns with our findings of a specificity of only 5% [13]. These indices, while useful, should be employed as part of a broader diagnostic strategy rather than relied upon in isolation.

The study provides a unique and thorough assessment of various hematological indicators, including the Green and King Index, RDW Index, and England and Fraser Index, primarily for distinguishing between IDA and TT. It introduces practical and cost-effective diagnostic tools by focusing on indices derived from basic complete blood count (CBC) tests. It also demonstrates the great clinical value of these measures through strong statistical validation. These findings are especially applicable for enhancing diagnostic precision in situations with limited resources, where access to modern diagnostic tools is frequently restricted. However, a limitation of the present study is the small sample size, which might limit the generalizability of the findings. Additionally, the study population was specific to a certain demography, which may not fully represent the variability in clinical presentations across different regions or ethnic groups.

To further advance research, it is important to authenticate the efficacy of these indicators in broader and more heterogeneous populations to guarantee their relevance in various contexts. Furthermore, the use of artificial intelligence (AI) in automated analyzers, like the Sysmex XN 1000, has the potential to greatly improve diagnostic precision and effectiveness. AI-driven algorithms can swiftly analyze large datasets and identify patterns. These algorithms can refine diagnostic criteria and provide predictive insights, improving early diagnosis and enabling personalized treatment plans [24,25].

## Conclusions

Accurate differentiation between IDA and TT is essential for effective treatment and prognosis. In resource-poor settings, hematological indices like the England and Fraser, RDW, and Green and King indices, derived from basic CBC tests, are crucial for distinguishing between IDA and TT. In the present study, these indices can facilitate prompt diagnosis in resource-limited environments.
